# Real-time tentative assessment of the epidemiological characteristics of novel coronavirus infections in Wuhan, China, as at 22 January 2020

**DOI:** 10.2807/1560-7917.ES.2020.25.3.2000044

**Published:** 2020-01-23

**Authors:** Peng Wu, Xinxin Hao, Eric H Y Lau, Jessica Y Wong, Kathy S M Leung, Joseph T Wu, Benjamin J Cowling, Gabriel M Leung

**Affiliations:** 1 World Health Organization (WHO) Collaborating Centre for Infectious Disease Epidemiology and Control, School of Public Health, Li Ka Shing Faculty of Medicine, The University of Hong Kong, Hong Kong Special Administrative Region, China; 2 These authors are joint senior authors with equal contribution

**Keywords:** Coronavirus, public health

## Abstract

A novel coronavirus (2019-nCoV) causing severe acute respiratory disease emerged recently in Wuhan, China. Information on reported cases strongly indicates human-to-human spread, and the most recent information is increasingly indicative of sustained human-to-human transmission. While the overall severity profile among cases may change as more mild cases are identified, we estimate a risk of fatality among hospitalised cases at 14% (95% confidence interval: 3.9–32%).

Four strains of coronaviruses are known to spread easily in humans, causing generally-mild acute respiratory illnesses known as the common cold [1]. A much larger number of coronaviruses have been detected in animals, particularly in bats, but have not been found in humans [2]. Prior to December 2019 when clusters of pneumonia cases with unknown aetiology were detected in Wuhan, China, only two additional strains of coronaviruses had caused outbreaks of severe acute respiratory disease around the world [3]. In the 2003 outbreaks of severe acute respiratory syndrome coronavirus (SARS-CoV) infections in mainland China, Hong Kong and a number of other locations, there were more than 8,000 documented cases and 774 deaths [4]. Since 2012, outbreaks of Middle East respiratory syndrome coronavirus (MERS-CoV) infection have occurred in the Middle East [5], and in 2015, there was a large outbreak in South Korea [6,7]. Super-spreading events have contributed to large outbreaks of SARS-CoV and MERS-CoV [8-10].

On 9 January 2020, a novel coronavirus, 2019-nCoV, was officially identified as the cause of an outbreak of viral pneumonia in Wuhan, China [11]. Wuhan is a large city of more than 11 million people located in central China around 1,200 km south of Beijing. As of 22 January, there have been 440 confirmed 2019-nCoV infections reported in 13 provinces and municipalities in mainland China and five other countries and regions overseas, with an increasing number of cases reported in recent days (Figure 1). Here, we describe the preliminary epidemiological characteristics of 2019-nCoV infections based on publicly-available information (including some media reports) before an official ‘line list’ of confirmed cases becomes available.

**Figure 1:**
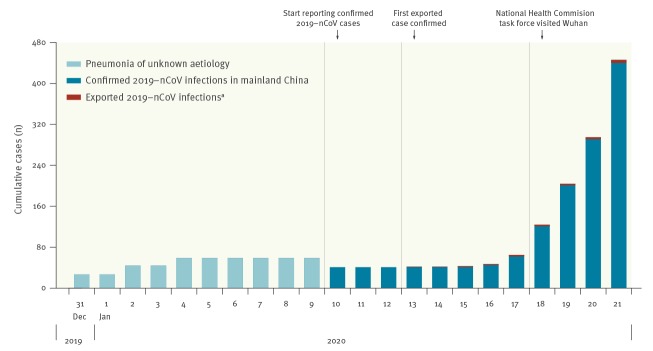
Increase in laboratory-confirmed cases of 2019-nCoV infection over time, as at 21 January 2020

nCoV: novel coronavirus.

^a^ Refers to 2019-nCov infections outside mainland China.

Figure based on the date of case announcement by the Wuhan Municipal Health Commission and then the from National Health Commission.

## Early outbreak of viral pneumonia of unknown aetiology as at 9 January

On 31 December 2019, the Wuhan Municipal Health Commission announced a cluster of cases of viral pneumonia of unexplained aetiology. The Southern China Seafood Wholesale Market in Wuhan was suspected to be related to the first 27 pneumonia cases without identified pathogenic agents that were reported in late December 2019 [12]. Most of the early cases were reportedly either shop owners, largely in the West District of the Southern China Seafood Wholesale Market, or people who visited the market before symptom onset. This market is a large open complex of 50,000 square metres including sections selling seafood, fresh meat, produce, other perishable goods, and a very wide variety of live wild animals for consumption. While wet markets selling such perishable food products are common in China, they usually do not sell such a wide variety of wild animals. Environmental disinfection of the Southern China Seafood Wholesale Market was initiated on 30 December 2019 and the market was closed on 1 January 2020 [13]. Contact tracing was initiated, and by 5 January, more than 160 close contacts of these cases were under medical surveillance, and none of them had an infection [14].

## Early information on 2019-nCoV infections as at 12 January

On 9 January 2020, a novel coronavirus, 2019-nCoV, was officially identified as the cause of the outbreak of pneumonia in Wuhan. Following the official announcement of the genetic sequence of the virus, on 11 January, 41 laboratory-confirmed cases of 2019-nCov infection with pneumonia were reported in Wuhan [15]. While the case definition for laboratory-confirmed cases has not been officially published, our understanding is that the initial case definition required (i) fever, (ii) x-ray evidence of pneumonia, (iii) white blood cell count normal or low or low lymphocyte count, (iv) antibiotic treatment for 3 days without improvement, in addition to (v) one or more recent visits to Wuhan or direct or indirect exposure to a wet market in Wuhan, and (vi) a respiratory specimen positive for 2019-nCoV and confirmed as 2019-nCoV by whole genome sequencing. The earliest known case had illness onset on 8 December 2019.

Between 10 and 12 January when there were no new cases reported, the Wuhan Municipal Health Commission announced that no new cases of 2019-nCoV infection were identified with illness onset after 3 January. As at 11 January, more than 700 close contacts were under medical surveillance, more than half being healthcare workers (HCWs), with no infections being identified [15]. Among the first 41 confirmed cases, approximately 70% reported exposure to the Southern China Seafood Wholesale Market [16]. Using the method described by Cauchemez et al. [17], assuming that the market was the only source of zoonotic infections, we estimated *R*_0_ to be 0.3 (95% confidence interval (95% CI): 0.17–0.44).

## Information on exported cases identified outside mainland China as at 17 January

On 13 January 2020, Thai health authorities reported an imported case in a person in their 60s who had travelled from Wuhan. This person had onset of an upper respiratory illness on 5 January and landed in Bangkok on 8 January, and was screened out by the thermal scanners at the airport. They did not visit the Southern China Seafood Wholesale Market, but reported visiting another wet market in Wuhan. On 16 January, Japanese health authorities reported a confirmed imported case in a person in their 30s who had travelled from Wuhan and landed in Kanagawa on 6 January. Illness onset was on 3 January and the person has since recovered. The person had not visited any wet market in Wuhan, but had visited a close relative who was in hospital in Wuhan with pneumonia. A third case was reported by Thai health authorities on 17 January. This person was in their 70s and had landed in Bangkok on 13 January, reporting an illness onset on 6 January and unclear history of exposure to the market in Wuhan. On 20 January, the fourth exported case from Wuhan, a person in their 30s visiting South Korea, was identified in Seoul. This person reported no previous visit to wet markets in Wuhan nor contact with any other case within 2 weeks before illness onset. The lack of exposure history to wet markets in Wuhan in two of four generally-mild exported cases indicated that there might be a larger number of undetected infections in Wuhan.

Two family clusters involving five people were reported from 15 to 16 January. One cluster of three close relatives all with illness onset on the same day, were thought to have occurred through a common exposure since they all lived together and worked in the same stall in the Southern China Seafood Wholesale Market. The other cluster involved a couple and may have occurred through human-to-human transmission with a serial interval of 5 days [18].

The basic reproduction number *R*_0_ is defined as the expected number of secondary cases produced by a typical single infection in a completely susceptible population. If there had been only one case infected by human-to-human transmission among the first 41 identified cases by that date, it implies *R*_0_ was 0.02 (i.e. 1/41) [19].

## Information on cases after creation of the National Health Commission task force on 18 January

On 19 January 2020, a task force under the National Health Commission, created on 18 January, visited Wuhan. On 20 January, 136 new infections in Wuhan, including 100 mild cases, 33 severe cases and three critically ill cases, were reported by the Wuhan Municipal Health Commission. Three traveller cases from Wuhan were also identified in Beijing and Shenzhen, possibly suggesting a large number of infections in Wuhan itself. However, further information on the most recently confirmed 2019-nCoV infections is required to determine the transmissibility of the virus and the severity profile of infections.

On 20 January, the task force reported three possible episodes of human-to-human transmission. This included, in two different families, family members who had not recently visited Wuhan being laboratory-confirmed with 2019-nCoV infection after other family members came back from Wuhan and were confirmed cases. A possible super-spreading event was also speculated by the task force given that there had been 15 HCWs infected with the virus in Wuhan until 20 January [20]. On 21 January, additional exported cases were reported in Taiwan and the United States, with illness onset on 11 and 19 January respectively.

## Risk of fatality among hospitalised cases

**Figure 2:**
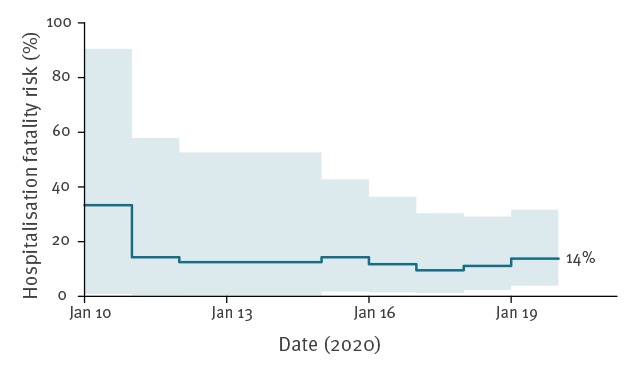
Temporal changes in estimated risk of death among laboratory-confirmed cases of 2019-nCoV admitted to hospital^a^, Wuhan, China, as at 21 January 2020

We estimated the hospital fatality risk, i.e. the risk of fatality among hospitalised cases [21] using the formula (fatal cases)/(fatal cases + recovered cases), which provides a more accurate early estimate of the hospital fatality risk compared with (fatal cases)/(all cases) [22]. We estimated the associated 95% CI for the hospital fatality risk using the binomial distribution. According to the update on 21 January 2020 when information on deaths and recoveries were reported, four cases had died while 25 had recovered, and our estimate of the hospital fatality risk is therefore 14% (95% CI: 3.9–32%). The estimate of the hospital fatality risk remained fairly stable over the 10 day period since the first death was announced on 11 January (Figure 2). If deaths continue to be reported without any corresponding increase in reported recoveries, the formula will overestimate the risk of fatality.

nCoV: novel coronavirus.

^a^ Based on the formula (fatal cases) / (fatal cases + recovered cases).

## Comparison of two hypothetical scenarios: one with large zoonotic spillover and one with small zoonotic spillover

To date, as at 22 January, official information has not been sufficient to determine the source of infection and risk of human-to-human transmission for the newly emerged 2019-nCoV. Two competing hypotheses are therefore proposed and compared as potential explanations for the characteristics of the current outbreak of 2019-nCoV infections within and outside mainland China. Scenario 1 comprises a large zoonotic spillover event starting in early December 2019, perhaps over a number of days or weeks, and very limited human-to-human transmission subsequently. Scenario 2 comprises a small zoonotic spillover event in early December 2019 followed by efficient human-to-human transmission. Evidence consistent or inconsistent with each of these two scenarios is outlined in the Table.

**Table:** Evidence on transmission dynamics of human infections with 2019-nCoV, as at 22 January 2020

Observation	Date	Interpretation	Supports Scenario 1?^a^	Supports Scenario 2?^b^
By 20 January, 198 laboratory-confirmed cases, 3 fatal, 25 recovered [28]	20 Jan 2020	Fatality risk among hospitalised cases is 11%^c^	Yes	Yes
No infections among the more than 700 people under medical surveillance, including HCWs [28]	20 Jan 2020	Very low human-to-human transmissibility	Yes	No (Note: assuming adequate contact tracing and surveillance)
Only one likely human-to-human cluster among the first 41 cases [18]	20 Jan 2020	Very low human-to-human transmissibility (*R*_0_ of 0.02)	Yes	No (Note: assuming adequate contact tracing)
Only approximately 70% of the first 41 cases had exposure to Southern China Seafood Wholesale Market [16]	15 Jan 2020	Low human-to-human transmissibility (*R*_0_ of 0.3)	Yes	No (Note: possible selection bias towards identifying cases linked to that market)
New cases with a travel history to Wuhan before onset were confirmed in other cities in China [29]	20 Jan 2020	Indicative of many mild to moderate infections (not requiring hospitalisation) in Wuhan	Yes	Yes
Four exported cases to other countries, all with relatively mild illness [30,31]	12–20 Jan 2020	Indicative of many mild to moderate infections not necessitating hospitalisation or outpatient medical care in Wuhan	Yes	Yes
Four exported cases, at least three of whom had no contact with Southern China Seafood Wholesale Market [30,31]	12–20 Jan 2020	At least some limited human-to-human transmission (unclear *R*_0_)	Yes	Yes
Two family clusters in Guangdong, with family members who did not visit Wuhan but were infected after the other family member(s) returning from Wuhan were confirmed with the infection [29]	20 Jan 2020	At least some limited human-to-human transmission (unclear *R*_0_)	Yes	Yes
15 HCWs confirmed with infection of 2019-nCoV (not clear whether infections were from one case or multiple cases) [20]	20 Jan 2020	Super-spreading event? Could still be consistent with limited human-to-human transmission if an isolated incident (unclear *R*_0_)	Yes	Yes
Exported cases identified in Taiwan and the United States with illness onset dates on 11 and 19 January [32,33]	22 Jan 2020	Could be because of surveillance bias, but is more consistent with an increase in incidence of infections over time	No (Note: possible selection bias because of enhanced surveillance towards identifying more recent cases)	Yes

HCWs: healthcare workers; nCoV: novel coronavirus; MHC: Municipal Health Commission.

^a^ Most infections are zoonotic with limited human-to-human transmission (*R*_0_ < 1).

^b^ Initial zoonotic spillover with efficient human-to-human transmission (*R*_0_ > 1).

^c^ Estimated as (fatal cases) / (fatal cases + recovered cases).

## Discussion

In this article, we describe a preliminary assessment of the outbreak of infections with the newly identified 2019-nCoV. This assessment is based on the cases of infection reported over time by health authorities in Wuhan and then at the national level, as well as the media in China and other countries. One of the most urgent priorities is to determine the degree of human-to-human transmissibility of the novel pathogen, and accordingly, this is where information is most urgently needed. We outline two possible scenarios in the Table  and find that the early evidence was most consistent with limited human-to-human transmissibility, however more recent data seem to be increasingly more compatible with scenario 2 in which sustained human-to-human transmission has been occurring. Determining the exposure profile among the recently confirmed cases would directly contribute to this assessment. Additional information on approaches to case identification and laboratory testing protocols in Wuhan and in other cities in China would also be informative. A separate priority is to identify the animal reservoir of this novel pathogen and any intermediary hosts, including potential supply chains of wild or game meat.

It is challenging to judge severity from the information available to date. We estimated the risk of death among hospitalised cases of around 14% (Figure 2). For both SARS-CoV and MERS-CoV infections, the risk of severe disease increases substantially with age and with the presence of underlying conditions [23-25]. One other caveat with estimating severity is that there can be long delays between hospitalisation and death for infections that are ultimately fatal. For SARS in Hong Kong, the average time from illness to death for fatal cases was 24 days [26]. This means that early estimates of the case fatality risk that ignore the potential outcomes of cases still in hospital are typically underestimates of the final severity profile [27]. We accounted for that by only including cases that either died or recovered in our estimate of the hospital fatality risk. Given that the cases reported outside Wuhan have mostly not been severe, it would be reasonable to infer that there might be a large number of undetected relatively mild infections in Wuhan and that the infection fatality risk is below 1% or even below 0.1%.

There are a number of limitations to our analyses. Most importantly, they are only based on data in the public domain to date. Detailed information has not yet been released by authoritative sources on the most recently reported cases. In our analysis in the Table, support for scenario 2 might increase if contact tracing or medical surveillance was incomplete, if there was incomplete ascertainment of clusters or if there was an early focus in testing cases linked to the Southern China Seafood Wholesale Market that led to a selection bias in the prevalence of market exposures among the early cases.

## References

[1] Su S Wong G Shi W Liu J Lai ACK Zhou J Epidemiology, Genetic Recombination, and Pathogenesis of Coronaviruses. Trends Microbiol. 2016;24(6):490-502. 10.1016/j.tim.2016.03.003 27012512 PMC7125511

[2] Fan Y Zhao K Shi ZL Zhou P Bat Coronaviruses in China. Viruses. 2019;11(3):E210. 10.3390/v11030210 30832341 PMC6466186

[3] Hui DS Epidemic and Emerging Coronaviruses (Severe Acute Respiratory Syndrome and Middle East Respiratory Syndrome). Clin Chest Med. 2017;38(1):71-86. 10.1016/j.ccm.2016.11.007 28159163 PMC7131795

[4] Guan Y Peiris JS Zheng B Poon LL Chan KH Zeng FY Molecular epidemiology of the novel coronavirus that causes severe acute respiratory syndrome. Lancet. 2004;363(9403):99-104. 10.1016/S0140-6736(03)15259-2 14726162 PMC7112497

[5] World Health Organization Regional Office for the (WHO/Eastern Mediterranean). MERS Situation Update. Cairo: WHO/Eastern Mediterranean; Nov2019. [Accessed 11 Jan 2020]. Available from: http://applications.emro.who.int/docs/EMRPUB-CSR-241-2019-EN.pdf?ua=1&ua=1

[6] Cowling BJ Park M Fang VJ Wu P Leung GM Wu JT Preliminary epidemiological assessment of MERS-CoV outbreak in South Korea, May to June 2015. Euro Surveill. 2015;20(25):7-13. 10.2807/1560-7917.ES2015.20.25.21163 26132767 PMC4535930

[7] Kim KH Tandi TE Choi JW Moon JM Kim MS Middle East respiratory syndrome coronavirus (MERS-CoV) outbreak in South Korea, 2015: epidemiology, characteristics and public health implications. J Hosp Infect. 2017;95(2):207-13. 10.1016/j.jhin.2016.10.008 28153558 PMC7114867

[8] Wong G Liu W Liu Y Zhou B Bi Y Gao GF MERS, SARS, and Ebola: The Role of Super-Spreaders in Infectious Disease. Cell Host Microbe. 2015;18(4):398-401. 10.1016/j.chom.2015.09.013 26468744 PMC7128246

[9] Kucharski AJ Althaus CL The role of superspreading in Middle East respiratory syndrome coronavirus (MERS-CoV) transmission. Euro Surveill. 2015;20(25):14-8. 10.2807/1560-7917.ES2015.20.25.21167 26132768

[10] Amer H Alqahtani AS Alzoman H Aljerian N Memish ZA Unusual presentation of Middle East respiratory syndrome coronavirus leading to a large outbreak in Riyadh during 2017. Am J Infect Control. 2018;46(9):1022-5. 10.1016/j.ajic.2018.02.023 29661625 PMC7115299

[11] World Health Organization (WHO). WHO Statement Regarding Cluster of Pneumonia Cases in Wuhan, China. Beijing: WHO; 9 Jan 2020. [Accessed 11 Jan 2020]. https://www.who.int/china/news/detail/09-01-2020-who-statement-regarding-cluster-of-pneumonia-cases-in-wuhan-china

[12] Wuhan Municipal Health Commission. [Update on outbreak of pneumonia cases in Wuhan]. 31 Dec 2019. [Accessed 13 Jan 2020]. Chinese. Available from: http://wjw.wuhan.gov.cn/front/web/showDetail/2019123108989

[13] Beijing News. [Southern China Seafood Market linked to unexplained pneumonia outbreak]. 2 Jan 2020. [Accessed 13 Jan 2020]. Chinese. Available from: http://epaper.bjnews.com.cn/html/2020-01/02/content_775695.htm

[14] Wuhan Municipal Health Commission. [Report on unexplained viral pneumonia]. 5 Jan 2020. [Accessed 21 Jan 2020]. Chinese. Available from: http://wjw.wuhan.gov.cn/front/web/showDetail/2020010509020

[15] Wuhan Municipal Health Commission. [Report on unexplained viral pneumonia]. 11 Jan 2020. [Accessed 21 Jan 2020]. Chinese. Available from: http://wjw.wuhan.gov.cn/front/web/showDetail/2020011109035

[16] Cheung J. Ill husband and wife add to Wuhan riddle. Hong Kong; The Standard; 16 Jan 2020. [Accessed 20 Jan 2020]. Available from: https://www.thestandard.com.hk/sections-news-print/215457/Ill-husband-and-wife--add-to-Wuhan-riddle

[17] Cauchemez S Epperson S Biggerstaff M Swerdlow D Finelli L Ferguson NM Using routine surveillance data to estimate the epidemic potential of emerging zoonoses: application to the emergence of US swine origin influenza A H3N2v virus. PLoS Med. 2013;10(3):e1001399. 10.1371/journal.pmed.1001399 23472057 PMC3589342

[18] Wuhan Municipal Health Commission. [Questions and answers on pneumonia due to novel coronavirus infection]. 14 Jan 2020. [Accessed 20 Jan 2020]. Chinese. Available from: http://wjw.wuhan.gov.cn/front/web/showDetail/2020011509040

[19] Cauchemez S Van Kerkhove MD Riley S Donnelly CA Fraser C Ferguson NM Transmission scenarios for Middle East Respiratory Syndrome Coronavirus (MERS-CoV) and how to tell them apart. Euro Surveill. 2013;18(24):20503. 23787162 PMC4088931

[20] World Health Organization Regional Office for the Western Pacific (WHO/Pacific). World Health Organization Western Pacific Twitter Feed. Manila: WHO/Pacific; 8:35 PM 20 Jan 2020. Available from: https://twitter.com/WHOWPRO

[21] Yu H Cowling BJ Feng L Lau EH Liao Q Tsang TK Human infection with avian influenza A H7N9 virus: an assessment of clinical severity. Lancet. 2013;382(9887):138-45. 10.1016/S0140-6736(13)61207-6 23803487 PMC3801178

[22] Ghani AC Donnelly CA Cox DR Griffin JT Fraser C Lam TH Methods for estimating the case fatality ratio for a novel, emerging infectious disease. Am J Epidemiol. 2005;162(5):479-86. 10.1093/aje/kwi230 16076827 PMC7109816

[23] Cowling BJ Muller MP Wong IO Ho LM Lo SV Tsang T Clinical prognostic rules for severe acute respiratory syndrome in low- and high-resource settings. Arch Intern Med. 2006;166(14):1505-11. 10.1001/archinte.166.14.1505 16864761

[24] Lau EH Hsiung CA Cowling BJ Chen CH Ho LM Tsang T A comparative epidemiologic analysis of SARS in Hong Kong, Beijing and Taiwan. BMC Infect Dis. 2010;10(1):50. 10.1186/1471-2334-10-50 20205928 PMC2846944

[25] Hui DS Azhar EI Kim YJ Memish ZA Oh MD Zumla A Middle East respiratory syndrome coronavirus: risk factors and determinants of primary, household, and nosocomial transmission. Lancet Infect Dis. 2018;18(8):e217-27. 10.1016/S1473-3099(18)30127-0 29680581 PMC7164784

[26] Leung GM Hedley AJ Ho LM Chau P Wong IO Thach TQ The epidemiology of severe acute respiratory syndrome in the 2003 Hong Kong epidemic: an analysis of all 1755 patients. Ann Intern Med. 2004;141(9):662-73. 10.7326/0003-4819-141-9-200411020-00006 15520422

[27] Jewell NP Lei X Ghani AC Donnelly CA Leung GM Ho LM Non-parametric estimation of the case fatality ratio with competing risks data: an application to Severe Acute Respiratory Syndrome (SARS). Stat Med. 2007;26(9):1982-98. 10.1002/sim.2691 16981181 PMC7169492

[28] Chinese National Health Commission (NHC). [Experts answer reporters' questions on pneumonia outbreak of new coronavirus infection]. Beijing: NHC; 20 Jan 2020. [Accessed 20 Jan 2020]. Chinese http://www.nhc.gov.cn/xcs/s7847/202001/8d735f0bb50b45af928d9944d16950c8.shtml

[29] Chinese National Health Commission (NHC). [Press conference in response to the outbreak of pneumonia caused by 2019-nCoV]. Beijing: NHC; 21 Jan 2020. [Accessed 21 Jan 2020]. Chinese. Available from: http://www.nhc.gov.cn/xcs/s7847/202001/8d735f0bb50b45af928d9944d16950c8.shtml

[30] World Health Organization (WHO). Novel Coronavirus – Thailand (ex-China). Geneva: WHO: 14 Jan 2020. Available from: https://www.who.int/csr/don/14-january-2020-novel-coronavirus-thailand/en/

[31] World Health Organization (WHO). Novel Coronavirus – Japan (ex-China). Geneva: WHO; 16 Jan 2020. Available from: https://www.who.int/csr/don/16-january-2020-novel-coronavirus-japan-ex-china/en/

[32] Centers for Disease Control and Prevention (CDC). First Travel-related Case of 2019 Novel Coronavirus Detected in United States. Atlanta: CDC; 21 Jan 2020. Available from: https://www.cdc.gov/media/releases/2020/p0121-novel-coronavirus-travel-case.html

[33] Taiwan Centers for Disease Control (CDC). Taiwan timely identifies first imported case of severe special infectious pneumonia from Wuhan, China through onboard quarantine; Central Epidemic Command Center (CECC) raises travel notice level for Wuhan, China to Level 3: Warning. Taipei City: CDC; 21 Jan 2020. Available from: https://www.cdc.gov.tw/En/Bulletin/Detail/pVg_jRVvtHhp94C6GShRkQ?typeid=158

